# Metabolic reprogramming of glioma-associated macrophages identifies detoxification and energetic macrophages as drivers of immunosuppression and therapeutic vulnerability

**DOI:** 10.3389/fimmu.2026.1752553

**Published:** 2026-02-11

**Authors:** Sujie Gu, Zhonghao Dou, Haoyu Shen, Hongfu Zhang, Yueyang Ba, Jianjun Gu, Peng Zhang

**Affiliations:** 1 Department of Neurosurgery, Henan Provincial People’s Hospital, Zhengzhou, Henan, China; 2 Department of Neurosurgery, People’s Hospital of Henan University, Zhengzhou, Henan, China; 3 Department of Neurosurgery, People’s Hospital of Zhengzhou University, Zhengzhou, Henan, China

**Keywords:** glioma-associated macrophages, machine learning, metabolism, prognostic model, tumor microenvironment

## Abstract

**Background:**

Glioma, a highly heterogeneous primary intracranial malignancy, features an immunosuppressive tumor microenvironment (TME) dominated by tumor-associated macrophages (TAMs). These glioma-associated macrophages (GAMs) critically drive disease progression, yet their metabolic reprogramming and clinical prognostic potential remain incompletely characterized. This study aimed to stratify GAMs by metabolic profiles and elucidate their clinical relevance, providing a framework for novel therapeutic strategies.

**Methods:**

We performed integrated multi-omics analysis of glioma single-cell RNA sequencing (scRNA-seq), bulk transcriptome sequencing, and clinical data. GAMs were stratified using metabolic pathway enrichment scores, and their abundance was correlated with patient prognosis. Supervised machine learning algorithms identified prognostic signature genes to construct a metabolic risk prediction model. Patients were stratified into high- and low-risk groups based on model-derived risk scores. Comprehensive profiling compared these groups across three dimensions: (i) dysregulated signaling pathways, (ii) tumor microenvironment characteristics, and (iii) genomic aberrations. Western blot (WB) analysis validated core gene expression in glioblastoma tumor tissues versus adjacent normal brain tissues.

**Results:**

This study reclassified GAMs into four metabolic subtypes—Glycolipid-Signaling (GSM), Detoxification and Energic (DEM), Polymetabolic (PmM), and Glycolipid Metabolism/Immunoregulatory (GMIM)—with DEMs exhibiting terminal differentiation, enrichment in detoxification/energy pathways, and significant correlation with advanced tumor grades and poor survival (p < 0.05). Machine learning leveraging DEM signature genes identified six core prognostic markers (CLIC1, FABP5, FCER1G, S100A8, S100A9, SPP1) and optimized a Stepwise Cox + Random Survival Forest model (C-index = 0.71). Applying this model, we identified high-risk gliomas exhibiting a paradoxical tumor microenvironment characterized by elevated immune cell infiltration and enhanced immunogenicity, yet impaired T-cell cytotoxicity. Concurrently, high-risk gliomas demonstrated hyperactivation of pro-tumorigenic pathways (e.g., mTOR, MAPK) and frequent EGFR amplification. Integration with EGFR amplification and IDH1 mutation status enhanced clinical prognostication. Western blot validation confirmed significant upregulation of all six core proteins in glioblastoma versus adjacent normal brain tissues.

**Conclusions:**

Metabolic subtyping identifies DEMs as critical drivers of glioma progression. The DEM-derived risk model, combined with EGFR/IDH status, provides a clinically actionable tool for prognosis and targeted therapy development.

## 1 Introduction

Gliomas, particularly glioblastoma (GBM), represent one of the most aggressive and lethal central nervous system malignancies, accounting for approximately 80% of primary malignant brain tumors (1). With an annual incidence of 3–5 cases per 100,000 individuals, GBM is characterized by inevitable recurrence, therapeutic resistance, and a dismal median survival of 12–15 months despite maximal multimodal therapy, underscoring the urgent need to unravel its pathobiological complexity (2, 3). The highly immunosuppressive and heterogeneous tumor microenvironment (TME) plays a pivotal role in fostering glioma progression, therapy resistance, and immune evasion, making it a critical focus for mechanistic and therapeutic exploration.

Within the glioma TME, tumor-associated macrophages (TAMs), comprising both brain-resident microglia and bone marrow-derived macrophages, constitute up to 30–50% of the cellular mass and play multiple pro-tumorigenic roles—supporting angiogenesis, remodeling extracellular matrix, and driving immunosuppression (4–6). Despite their dominance, the metabolic heterogeneity of TAMs remains poorly characterized. Emerging evidence suggests that metabolic reprogramming (e.g., glycolysis, oxidative phosphorylation, and lipid metabolism) underpins TAM functional plasticity (7). However, comprehensive classifications of TAM metabolic subtypes—particularly their clinical relevance in gliomas—remain incompletely characterized. Single-cell RNA sequencing (scRNA-seq) offers improved resolution over traditional approaches to dissect this heterogeneity, enabling the identification of spatially and temporally distinct TAM subpopulations while mapping their metabolic dependencies and crosstalk with tumor cells (8).

Concurrently, genomic aberrations in gliomas, such as somatic single-nucleotide polymorphisms (SNPs) and copy number variations (CNVs), drive oncogenic signaling pathways and intratumoral heterogeneity (9, 10). Recurrent alterations such as EGFR amplification, IDH1/2 mutation, and PTEN deletion not only define molecular subtypes but also shape the TME by modulating cytokine secretion and immune cell recruitment (9). The interplay between somatic SNPs (e.g., TP53 missense mutations) and CNVs (e.g., chromosome 7 gain/10 loss) may further amplify oncogenic signaling and influence therapeutic vulnerability (10). However, integrative analysis linking these genomic events to TME dynamics, particularly TAM metabolic states, remains underinvestigated.

In this study, we performed integrated analysis of glioma scRNA-seq, bulk sequencing data, and clinical profiles to define metabolic subtypes of glioma-associated macrophages (GAMs) and their clinical relevance. Focusing on the Detoxification and Energetic Macrophage (DEM) subtype, we leveraged DEM-specific signature genes to construct a prognostic risk model using machine learning. Subsequent comparative analysis of high- and low-risk cohorts revealed distinct patterns in: (i) tumor microenvironment dynamics, (ii) oncogenic pathway activation, and (iii) genomic alterations. Furthermore, we experimentally validated the differential expression patterns of core risk model genes between glioblastoma tumor tissues and adjacent normal brain tissues. This integrated framework establishes a novel metabolic-immune axis for glioma risk stratification and uncovers potential therapeutic vulnerabilities in high-risk glioma subtypes.

## 2 Materials and methods

### 2.1 Single-cell sequencing data acquisition and analysis

The single-cell RNA sequencing dataset (accession number: GSE182109) was retrieved from the Gene Expression Omnibus (GEO) database (https://www.ncbi.nlm.nih.gov/gds). For subsequent analyses, we selected 10 newly diagnosed glioma samples, comprising 6 glioblastomas (GBMs; GSM5518633, GSM5518634, GSM5518635, GSM5518636, GSM5518637, GSM5518639) and 4 low-grade gliomas (LGGs; GSM5518630, GSM5518631, GSM5518632, GSM5518638).

Raw sequencing data were processed using the R package Seurat (v4.4.0) for quality control and integration. Cells were filtered based on the following criteria: number of detected genes (nFeature_RNA) > 200, mitochondrial gene content (percent.mt) < 20%, and ribosomal gene content (percent.Ribo) < 50%. Potential doublets were identified and excluded using the scDblfinder algorithm prior to downstream analysis. Global normalization was performed using the SCTransform function in Seurat, followed by batch effect correction at the sample level via the Harmony algorithm to ensure cross-sample comparability. Dimensionality reduction was conducted using principal component analysis (PCA), and uniform manifold approximation and projection (UMAP) embedding was generated with the RunPCA, RunUMAP, FindNeighbors, and FindClusters functions (parameters: dims = 1:40, resolution = 0.2). Cell type annotation was performed using SingleR based on canonical marker genes.

To identify myeloid subpopulation-specific marker genes, the FindAllMarkers function in Seurat was employed with stringent thresholds: absolute log_2_ fold change (|log_2_FC|) ≥ 0.25, minimum percentage of cells expressing the gene in either cluster (min.pct) ≥ 0.1, and Benjamini–Hochberg adjusted p-value < 0.05.

### 2.2 Bulk sequencing and clinical data acquisition and preprocessing

Clinical metadata and transcriptomic profiles of glioblastoma (GBM) and low-grade glioma (LGG) patients were obtained from multiple public datasets as follows:

The Cancer Genome Atlas (TCGA) (https://tcga-data.nci.nih.gov/tcga/): Level 3 RNA sequencing data and corresponding clinical annotations were downloaded, yielding 658 tumor samples after excluding cases with missing survival time/status or survival time ≤ 0 days. Somatic single-nucleotide variant (SNV) data were retrieved from TCGA and processed using Mutect2 software, resulting in 644 overlapping genes for downstream analysis. Copy number variation (CNV) data were acquired via the R package “TCGAbiolinks”.

Gene Expression Omnibus (GEO) dataset GSE43378: Transcriptomic and clinical data were downloaded from GEO, with 50 tumor samples retained after excluding cases lacking overall survival (OS) data or with OS ≤ 0 days.

Chinese Glioma Genome Atlas (CGGA) (http://www.cgga.org.cn/): RNA sequencing data from three CGGA cohorts (CGGA301, CGGA325, CGGA693) were retrieved. Samples were excluded if they lacked survival/follow-up information or had OS ≤ 0 days, resulting in 285, 313, and 567 qualified tumor samples, respectively.

### 2.3 Trajectory, stemness, and metabolic pathway analysis

Developmental trajectories of macrophages were reconstructed using the Monocle2 algorithm with input as a normalized, scaled UMI count matrix from the Seurat subset; a CellDataSet object was initialized via newCellDataSet (expression family: negbinomial.size), followed by dimensionality reduction, pseudotime ordering, and trajectory inference with default parameters. Stemness and differentiation potential of macrophage subpopulations were evaluated using the “CytoTRACE2” R package, while metabolic pathway activity across subtypes was quantified via the “scMetabolism” package, which calculates enrichment scores based on single-cell transcriptomic profiles.

### 2.4 Overview of the risk model construction workflow

The overall workflow for constructing the integrative risk model is summarized as follows. First, univariate Cox regression analysis was performed in the TCGA-CRC cohort to identify prognostic RNAs. Second, these candidate RNAs were subjected to 10 machine learning–based survival algorithms and 101 algorithm combinations to build predictive models under a leave-one-out cross-validation framework. Third, all models were independently validated in other external GEO cohorts, and their prognostic performance was evaluated using Harrell’s concordance index (C-index). Finally, the model achieving the highest average C-index across all validation datasets was selected as the optimal risk signature and applied to stratify patients into high- and low-risk groups.

### 2.5 Risk score calculation based on six core genes

The RSF model was trained using the expression levels of the six core genes as predictors. For an individual *i* with six-gene feature vector $X_{i}=(x_{i1},\ldots ,x_{i6})$, the RSF produces an ensemble prediction by averaging over Bsurvival trees. The risk score was defined as the RSF-predicted scalar mortality (predicted risk), obtained from the randomForestSRC prediction output:


$${\text{RiskScore}}_{i}\equiv \hat{r}(X_{i}),$$

where $\hat{r}(\cdot )$ is the trained RSF mapping from the six-gene expression profile to a scalar risk value. In practice, $\hat{r}(X_{i})$ was calculated as predict(fit, new_data)$predicted. Higher risk scores indicate higher predicted mortality risk.

### 2.6 Mutation landscape analysis

Genomic alterations, including recurrent copy number variations (amplifications and deletions), were identified using GISTIC 2.0. Tumor mutational burden (TMB) and gene mutation frequencies were calculated with the R package “maftools”.

### 2.7 Tumor microenvironment heterogeneity analysis

To comprehensively characterize the TME, multiple immune infiltration algorithms were applied using the “IOBR” package to quantify the infiltration levels of distinct immune cell subsets across samples.

### 2.8 Clinical sample collection

This study was approved by the Ethics Committee of Henan Provincial People’s Hospital (Approval No.: 2025-116-02), and written informed consent was obtained from all participants. A total of 6 patients with pathologically confirmed glioblastoma were enrolled, with adjacent non-tumor brain tissue serving as the control group. All tissue samples were immediately snap-frozen in liquid nitrogen to preserve RNA and protein integrity for downstream molecular analyses.

### 2.9 Western blot

The proteins were isolated by RIPA lysis buffer (Beyotime, P0013B), and the concentrations were determined by the BCA detection kit (Beyotime, P0010). About 30μg proteins was loaded and electrophoresed onto 8% SDS polyacrylamide gel (GE Healthcare Bioscience, 30166428) and then transferred to polyvinylidene fluoride (PVDF) membranes (Millipore, IPVH00010). The membranes were blocked with 5% non-fat milk for 60min at 37 °C and were then incubated with primary antibodies against CLIC1 (1:1000, Thermo-Invitrogen, PA5-109551), FABP5 (1:1000, Thermo-Invitrogen, PA5-76920), FCER1G (1:1000, Thermo-Invitrogen, PA5-115222), S100A8 (1:1000, abclonal, A12018), S100A9 (1:3000, abclonal, A26782PM), SPP1 (1:1000, Thermo-Invitrogen, MA5-49933), and β-Tublin (1:5000, abmart, m20005) overnight at 4 °C, anti-rabbit secondary antibodies (1:5000, Jackson ImmunoResearch, 111-035-003) at room temperature for 2h. The detection of signal was conducted using Bryo ECL Kit (Beyotime, P0020). Band densitometry for Western blot was performed using ImageJ software (v1.53t, NIH).

### 2.10 Statistical analysis

Data processing, statistical analyses, and visualization were performed using R software (version 4.2.0). Subtype-specific overall survival (OS) was estimated and compared using the Kaplan–Meier method with the log-rank test. For continuous variables, differences between two groups were assessed by the Wilcoxon rank-sum test (non-parametric) or Student’s t-test (parametric), as appropriate. Categorical variables were compared using the chi-square test or Fisher’s exact test, depending on cell count thresholds. Multiple comparisons were corrected using the false discovery rate (FDR) method to adjust p-values. Associations between variables were evaluated via Pearson’s correlation analysis. All p-values were two-tailed, and statistical significance was defined as p < 0.05.

## 3 Results

### 3.1 Metabolic phenotyping of GAMs

**Figure 1:**
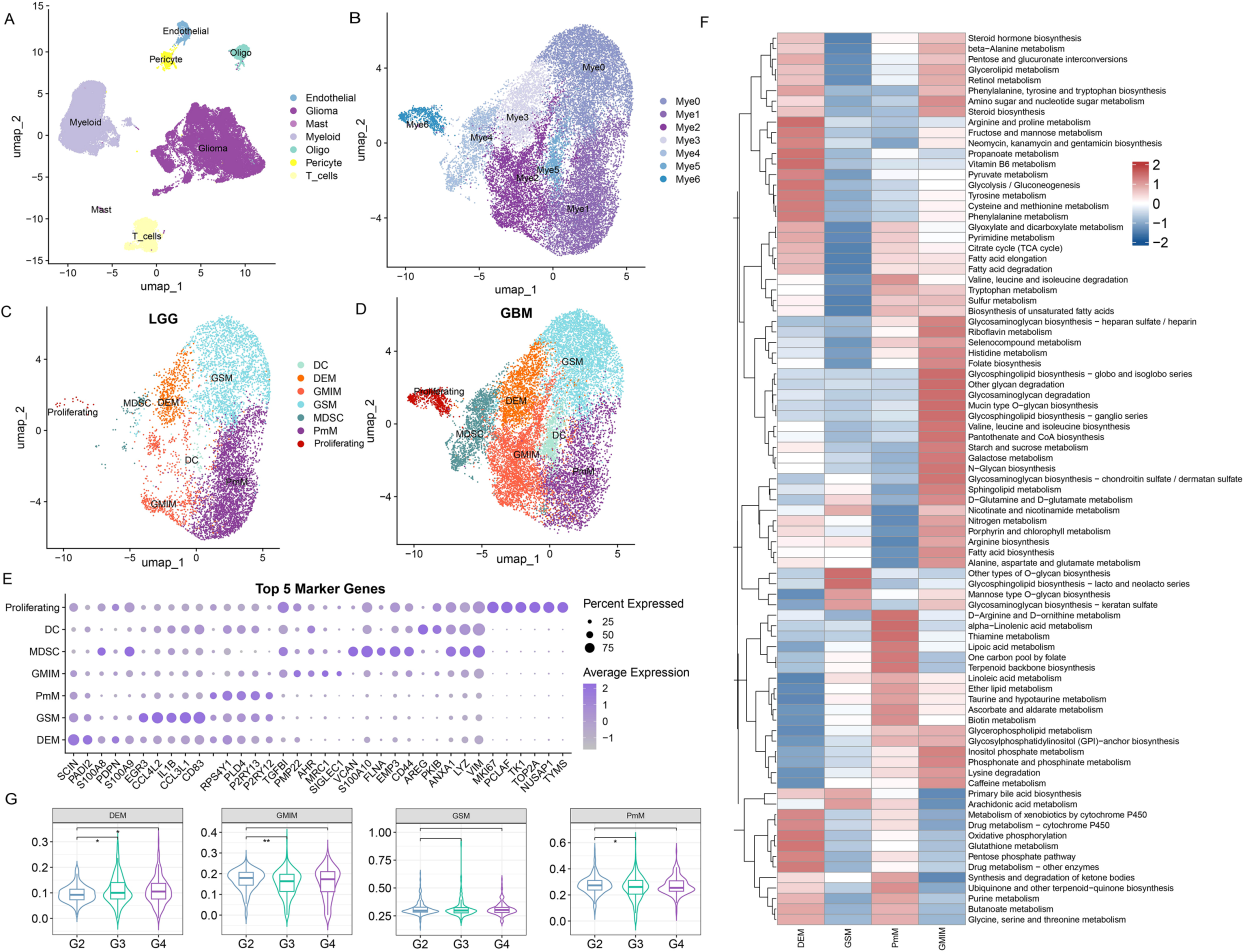
Metabolic subtyping and quantitative profiling of glioma-associated macrophages across WHO grades. **(A)** scRNA-seq analysis of GSE182109, UMAP projection of 54,993 single cells, colored by graph-based cell clusters and inferred cell types. **(B)** Subclassification of myeloid lineage cells with metabolic nomenclature. **(C, D)** Distribution of myeloid subtypes in low-grade glioma (LGG) **(C)** and glioblastoma (GBM) **(D)** patients. **(E)** The top five marker genes defining each myeloid subtype were ranked by expression percentage and average expression. **(F)** Heatmap depicting the metabolic pathway enrichment profiles of the four macrophage subtypes. **(G)** Proportional abundance of metabolic macrophage subtypes stratified by WHO grade. *p<0.05; **p<0.01.

After screening, accusation, removal of double cells, normalization processing, and dimensionality reduction analysis, a total of 54,993 cells were obtained and divided into 15 subgroups (**Supplementary Figures S1**-**S3**). Next, we annotated cell types on the basis of classical cell markers via “single R” and identified 7 principal cell types, namely, endothelial, glioma, mast, myeloid, oligo, pericyte and T cells (**Figure 1A**). To precisely define the heterogeneity and function of myeloid cells in gliomas, we subclustered myeloid cells into 7 clusters, including Mye 0–6 (**Figure 1B**). As shown in **Figure 1F**, macrophage subpopulations were classified into four metabolic phenotypes via scMetabolism pathway analysis, with nomenclature derived from dominant functional programs:

- Mye0: Glycolipid-Signaling Macrophages (GSM), defined by glycolipid-centered metabolism (glycolysis/gluconeogenesis, fructose-mannose pathways, glycero-/sphingolipid biosynthesis), providing substrates for inflammatory signal transduction.
- Mye1: Polymetabolic Macrophages (PmM), exhibiting multiplexed pathway activation (branched-chain amino acid degradation, pentose phosphate flux, purine/pyrimidine synthesis), reflecting an undifferentiated state with high plasticity.
- Mye2: Glycolipid Metabolism and Immunoregulatory Macrophages (GMIM), combining glycolipid processing with immune-regulatory functions (glycosaminoglycan biosynthesis, arachidonic acid metabolism), implicating roles in immunosuppression and tissue homeostasis.
- Mye3: Detoxification and Energetic Macrophages (DEM), specialized in coupled bioenergetics and detoxification (OXPHOS, TCA cycle, fatty acid β-oxidation, glutathione metabolism, CYP450-mediated xenobiotic clearance) for stress adaptation.

Our metabolic classification revealed four distinct macrophage phenotypes, representing glycolipid-driven signaling, polymetabolic plasticity, immunoregulatory metabolism, and detoxification-energetic programs, highlighting the functional heterogeneity of myeloid cells beyond classical polarization models.

The distributions of the four macrophage phenotypes among all single cells, GBM and LGG are shown in **Figures 1C, D**. Single-cell distribution density plots and colorimetric cluster profiling revealed significantly greater proportions of both the DEM and GMIM subtypes in GBM than in LGG, with GBM samples exhibiting denser clustering and distinct color-coded enrichment patterns for these macrophage subsets. The top five most biologically significant markers per subpopulation, ranked by their average log_2_FC and adjusted p values, were visualized via a heatmap to delineate subset-specific expression patterns (**Figure 1E**). To resolve macrophage subtype abundance at the bulk transcriptome level, we applied the bisqueRNA algorithm, a partial least squares-based deconvolution framework, to TCGA RNA-seq data, enabling robust estimation of the relative proportions of the four macrophage subtypes (GSM, DEM, PmM, and GMIM) across glioma samples. While GSM abundance remained consistent across glioma grades, DEM, GMIM, and PmM were elevated in grade III versus grade II tumors (p <0.05). Critically, only DEM showed further enrichment in grade IV gliomas (p <0.05), implicating its stage-specific role in disease progression.

### 3.2 Characterization of GAM subtypes

To evaluate the prognostic impact of the four macrophage phenotypes, Kaplan-Meier (KM) survival analyses were performed separately for WHO grade 2–3 (G2+G3) and grade 4 (G4) gliomas, stratified by macrophage subtype abundance. In grade 2–3 gliomas, high abundance of all four metabolic subtypes predicted significantly reduced median overall survival (mOS) (DEM: 5.25 vs. 8.19 years; GSM: 6.25 vs. 7.77 years; PmM: 5.48 vs. 7.96 years; GMIM: 5.25 vs. 7.88 years; all p < 0.05). In grade IV tumors, high abundance of DEM, GSM, and GMIM remained independent adverse predictors (DEM: 0.88 vs. 1.25 years, p = 0.0011; GSM: 0.94 vs. 1.24 years, p = 0.046; GMIM: 0.92 vs. 1.25 years, p = 0.0029), whereas PmM abundance showed no prognostic significance (1.11 vs. 1.15 years; p = 0.43). Critically, DEM and GMIM demonstrated universal cross-grade prognostic value, establishing these macrophage subtypes as key drivers of glioma progression.

**Figure 2:**
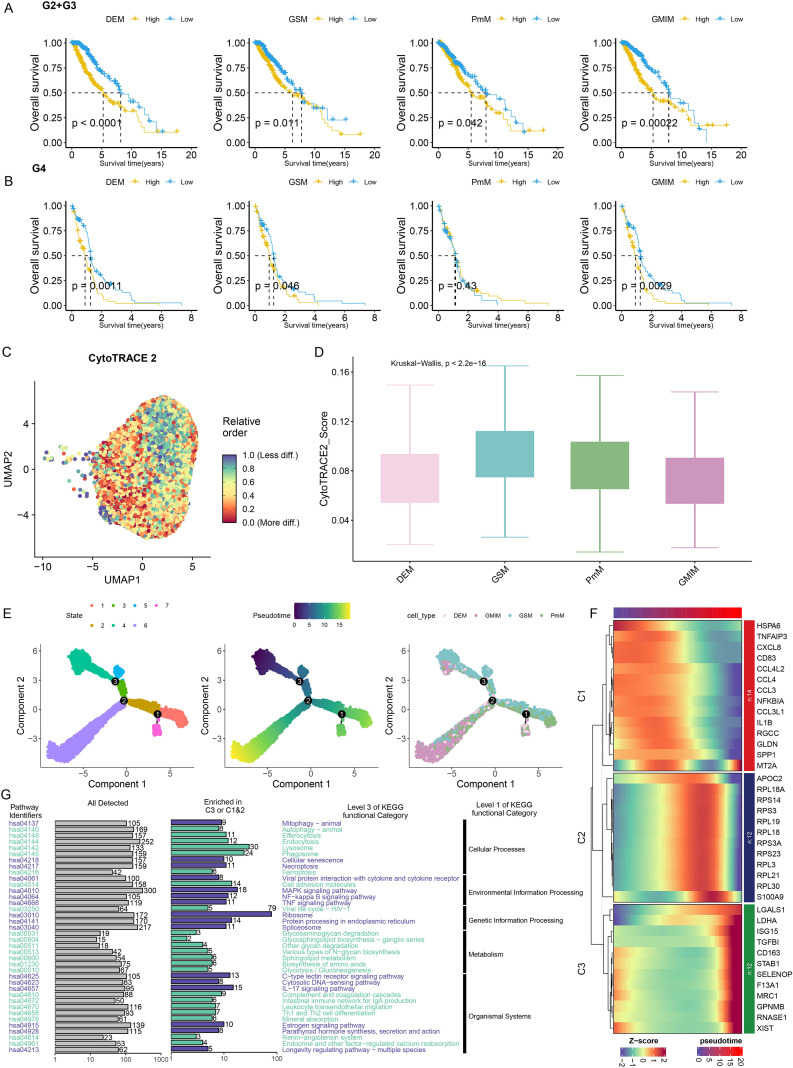
Characterization of four metabolic subtypes of glioma-associated macrophages. **(A, B)** Kaplan–Meier curve of overall survival in WHO grade 2–3 (G2+G3) **(A)** and grade 4 (G4) **(B)** gliomas on the basis of the abundances of four macrophage subtypes. **(C)** Stemness prediction of myeloid lineage cells via “CytoTRACE2”. **(D)** CytoTRACE scores of the four macrophage subtypes (p<2.2e-16). **(E)** Principal component analysis (PCA) plots of cell states, pseudotime, and cell types. **(F)** Temporal gene expression in macrophage subtypes. C1, C2, and C3 designate early-, mid-, and late-stage coexpression modules derived from pseudotime analysis, with adjacent genes representing signature markers for each respective module (ranked by fold change and adjusted p value). **(G)** KEGG pathway enrichment analysis of signature genes defining pseudotemporal modules: C3 (late-stage) versus combined C1/C2 (early/mid-stage). Navy blue denotes C3, whereas yellow represents the combined C1/C2.

The stemness potential of the macrophage subtypes was predicted using CytoTRACE2, an algorithm that infers cellular differentiation states from transcriptomic diversity. GSM exhibited the highest stemness score, followed by PmM, while GMIM and DEM displayed the lowest scores, indicating a more differentiated phenotype (**Figures 2C, D**). Pseudotime trajectory analysis using Monocle further supported this differentiation hierarchy, positioning GMIM and DEM at the terminal branches of the trajectory (**Figure 2E**).

### 3.3 Temporal resolution of differentially expressed genes across subtypes identified three co-expression modules, with the top 38 signature genes (ranked by fold change and adjusted p-value) showing stage-specific expression

C1, C2, and C3 co-expression modules mapped to early, intermediate, and terminal differentiation stages of GAMs, respectively (**Figure 2F**). Critically, the C3 module—enriched in terminally differentiated DEM and GMIM subtypes—showed significant functional enrichment in pro-inflammatory and stress-response pathways, including ribosome biogenesis, MAPK signaling, IL-17 signaling. In contrast, C1/C2 modules (expressed in less differentiated subtypes) were associated with lysosomal activity, phagocytosis, cell adhesion molecule signaling. These findings establish a differentiation-linked functional hierarchy wherein terminal GAM subsets (DEM/GMIM) acquire pro-inflammatory properties through C3 module activation, while early-stage subsets maintain homeostatic functions via C1/C2 modules (**Figure 2G**). Prognostic model construction based on DEM signatures.

Given the grade-dependent enrichment of DEMs and their consistent association with poor prognosis, we prioritized DEM subtypes for prognostic model development. Univariate Cox regression was first performed on the 71 DEM signature genes across five bulk RNA-seq cohorts (TCGA_GBM, GSE43378, CGGA_693, CGGA_301, and TCGA_LGG) to identify genes significantly correlated with overall survival (p < 0.05; Additional file 1). From these, 14 genes (S100A9, S100A8, PDPN, SPP1, FABP5, FCER1G, TYMP, LGALS1, FBP1, S100A11, ENO1, CLIC1, SH3BGRL3, LDHA) demonstrating prognostic consistency across all datasets were selected as candidate features for model training (**Supplementary Figure S4**).

**Figure 3:**
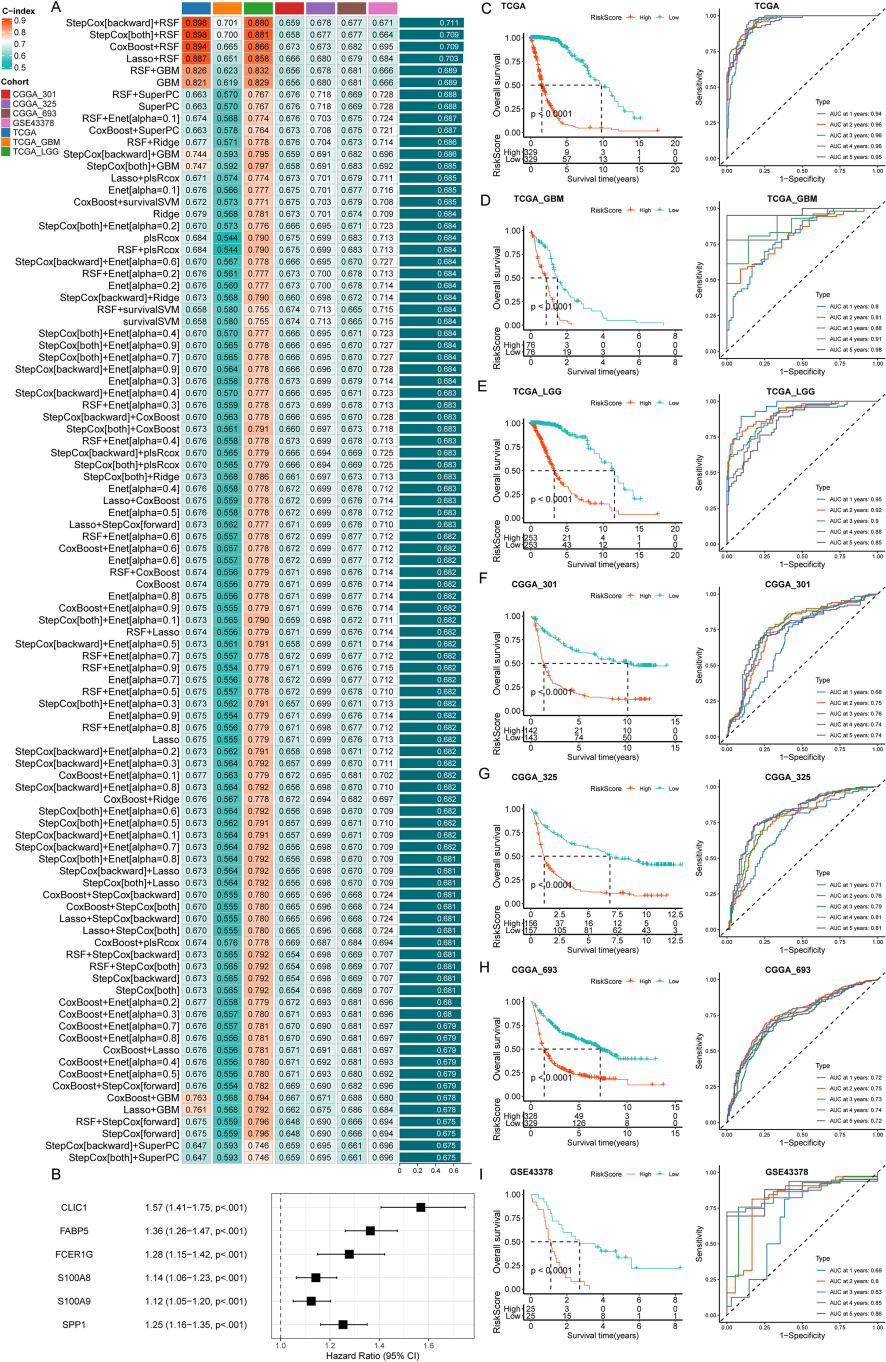
Prognostic model development and validation for gliomas based on DEM subtypes. **(A)** Prediction models via machine learning based on 14 prognostic genes. **(B)** Forest plot of univariate Cox analysis for the 6 key genes. **(C-I)** Predictive risk scores for both the training and validation sets in all cohorts.

To develop a robust glioma prognostic model, we integrated these 14 genes using a machine learning framework applied to the combined TCGA-GBM/LGG cohort. Specifically, 101 prediction models were fitted via leave-one-out cross-validation (LOOCV), and model performance was evaluated by calculating the concordance index (c-index) across validation datasets (**Figure 3A**). The optimal model—Stepwise Cox regression (backward elimination) combined with a Random Survival Forest (StepCox [backward] + RSF)—yielded the highest mean c-index (0.711) in the test set. This model was further refined to six key genes: CLIC1, FABP5, FCER1G, S100A8, S100A9, and SPP1 (**Figure 3B**).

Risk scores derived from the six-gene model were validated across all cohorts, with consistent stratification of patients into high- and low-risk groups (**Figures 3C–I**). In every dataset, the high-risk group exhibited significantly worse overall survival compared to the low-risk group (*p* < 0.001), with the mOS of high- vs low-risk as follows: TCGA 1.54 vs 9.78 years, TCGA_GBM 0.86 vs 1.47years, TCGA_LGG 3.24 vs 11.59 years, CGGA_301 1.36 vs 10.07 years, CGGA_325 1.16 vs 6.85years, CGGA_693 1.39 vs 7.21years and GSE43378 1.11 vs 2.71 years, respectively. Time-dependent receiver operating characteristic (ROC) analysis further confirmed robust predictive performance, with 1-, 2-, 3-, 4-, and 5-year area under the curve (AUC) values ≥ 0.68 across cohorts.

### 3.4 Comparative profiling of the tumor immune microenvironment in high- versus low-risk cohorts

**Figure 4:**
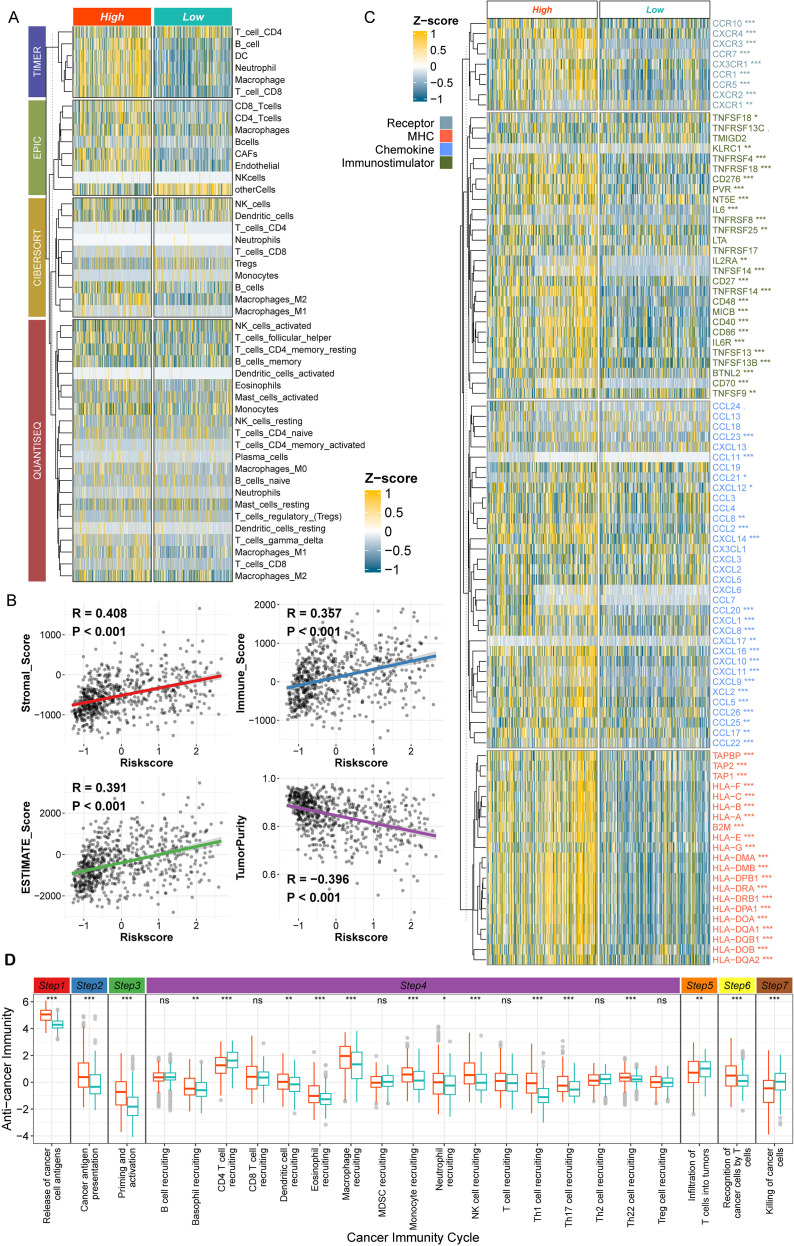
Comparative profiling of the tumor immune microenvironment in high- versus low-risk cohorts. **(A)** Immune infiltration analysis of the high- and low-risk groups via TIMER, EPIC, CIBERSORT, and QANTISEQ. **(B)** Correlation analysis between the risk score and immune infiltration (stromal score, immune score, estimate score, and tumor purity). **(C)** Differentially expressed immune factors between the high- and low-risk groups of gliomas. **(D)** Comparison of immune circulation activity via the TIP algorithm. The high-risk group is denoted by brick red, and the low-risk group is denoted by light green. *p<0.05; **p<0.01; ***p<0.001.

To characterize tumor immune microenvironment (TIME) heterogeneity between high- and low-risk groups, we systematically quantified immune cell infiltration patterns using four independent computational algorithms: TIMER, EPIC, CIBERSORT, and QUANTISEQ (11–14). Deconvolution analysis via TIMER and EPIC revealed significantly higher infiltration of macrophages, B cells, neutrophils, cancer-associated fibroblasts (CAFs), and CD8+ T cells in the high-risk group compared to the low-risk group. Notably, granular immune cell classification via CIBERSORT and QUANTISEQ further identified M2-polarized macrophages as the predominantly enriched subtype in high-risk tumors, with other cellular populations showing minimal intergroup variation (**Figure 4A**). This dichotomy suggests that M2 macrophage infiltration represents a dominant immunophenotypic hallmark of high-risk gliomas.

Consistent with these findings, immune infiltration scores calculated via the ESTIMATE algorithm demonstrated positive correlations between risk scores and stromal score, immune score, and ESTIMATE score, alongside a negative correlation with tumor purity (p < 0.01; **Figure 4B**). To dissect the molecular mechanisms underlying TIME divergence, we compared the expression profiles of key immune regulatory factors—including immune checkpoints, MHC molecules, chemokines, and immunostimulatory cytokines—between groups. High-risk tumors exhibited upregulated expression of multiple pro-inflammatory/immune-modulatory families, including CXCL, TNFSF, CCL, and HLA genes (**Figure 4C**).

To further interrogate functional TIME dynamics, we analyzed cancer-immunity cycle activity using the TIP algorithm (15). Compared to the low-risk group, high-risk gliomas displayed significantly enhanced activation in the early phases of the cycle: (1) release of cancer cell antigens, (2) cancer antigen presentation, (3) priming and activation of T cells, (4) trafficking of immune cells to tumors, and (6) recognition of cancer cells by T cells. In striking contrast, two critical downstream effector phases were markedly attenuated in high-risk tumors: (5) infiltration of T cells into the tumor parenchyma and (7) killing of cancer cells (**Figure 4D**).

Synthesizing these findings, our risk-stratified profiling uncovers a paradoxical tumor immune microenvironment (TIME) in high-risk gliomas: despite heightened immune infiltration and hyperactivated early immunogenic responses (antigen presentation/T cell priming), these tumors exhibit profoundly attenuated effector functions—specifically impaired intratumoral T cell infiltration and cytotoxic killing. This discordance mechanistically underpins adverse prognosis, establishing dysfunctional TIME dynamics as a hallmark of glioma aggression.

### 3.5 Functional annotation of risk-associated pathways in glioma

**Figure 5:**
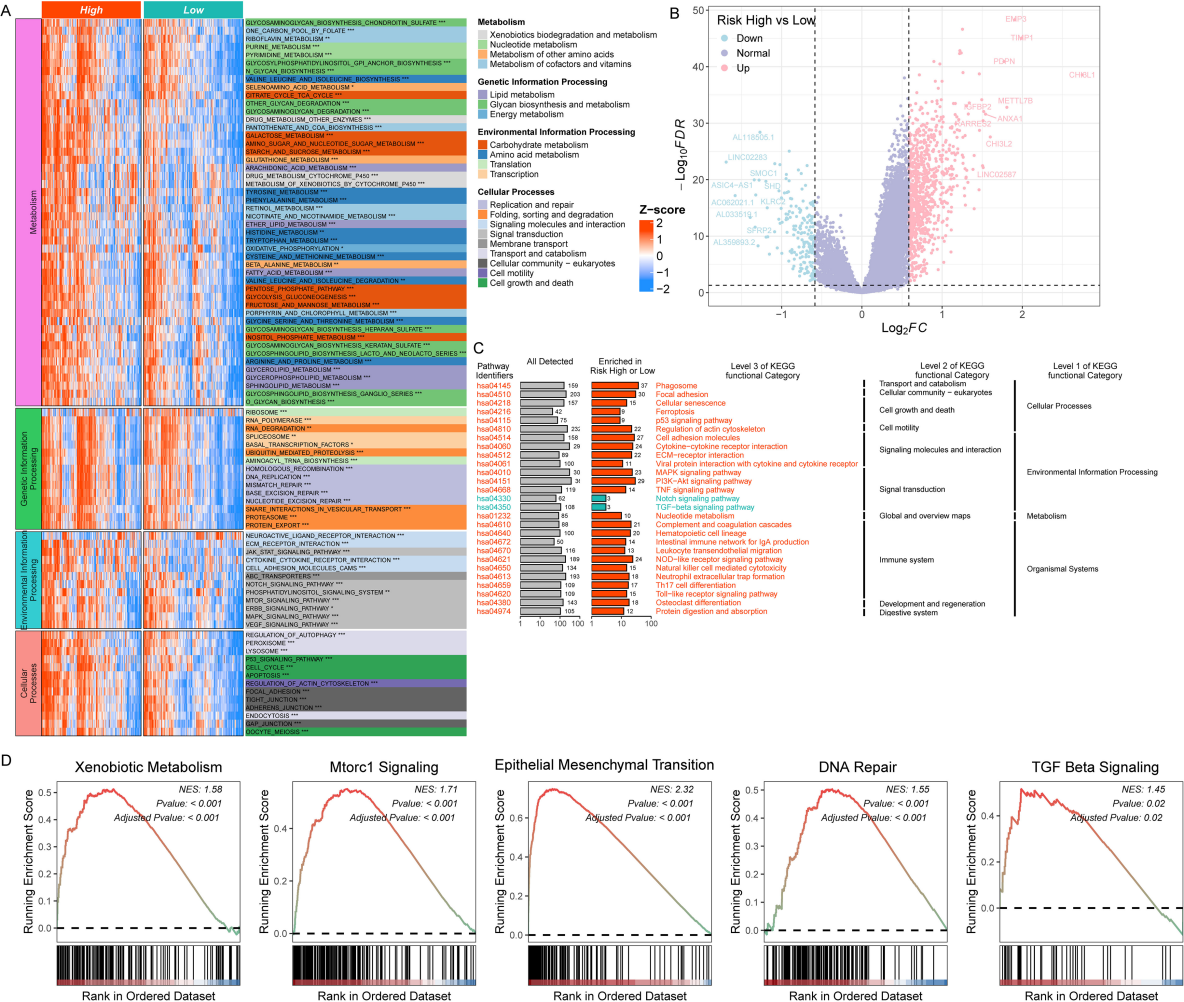
Functional annotation of risk-associated pathways in glioma. **(A)** Heatmap showing the differential enrichment of pathways between high- and low-risk gliomas in four functional modules: metabolism, genetic information processing, environmental information processing, and cellular processes. **(B)** Volcano plot illustrating differential gene expression between the high-risk and low-risk glioma groups (/log_2_FC/> 0.585 and p < 0.05). **(C)** Heatmaps showing the enriched pathways for upregulated and downregulated genes in high-risk gliomas. **(D)** Plots showing the GSEA of selected pathways related to glioma progression based on the risk coefficient. *p<0.05; **p<0.01; ***p<0.001.

To elucidate the molecular mechanisms driving tumor progression in high-risk gliomas, we performed Gene Set Variation Analysis (GSVA) to compare pathway enrichment profiles between high- and low-risk cohorts across four KEGG functional modules: metabolism, genetic information processing, environmental information processing, and cellular processes. Significant divergence was observed in all modules (**Figure 5A**).

Within the metabolism module, high-risk tumors exhibited enriched activity in pathways associated with xenobiotic and drug metabolism, including “Drug Metabolism - Other Enzymes”, “Drug Metabolism - Cytochrome P450”, and “Metabolism of Xenobiotics by Cytochrome P450”. In the cellular processes module, pathways linked to cell survival, proliferation, and stress responses were differentially regulated: high-risk gliomas showed upregulation of “Regulation of Autophagy”, “p53 Signaling Pathway”, “Focal Adhesion”, and “Apoptosis”. For genetic information processing, high-risk tumors displayed enhanced enrichment in “DNA Replication” and “Mismatch Repair”, while environmental information processing pathways such as “mTOR Signaling Pathway”, “MAPK Signaling Pathway”, and “VEGF Signaling Pathway” were significantly activated.

To identify key molecular drivers underlying these pathway differences, we performed differential gene expression analysis between risk groups using thresholds of |log_2_(fold change)| > 0.58496 and p < 0.05. This yielded 1,118 differentially expressed genes (DEGs), with 929 upregulated and 189 downregulated in the high-risk cohort (**Additional file S2**). The top five upregulated DEGs in high-risk gliomas were CHI3L1, TIMP1, EMP3, METTL7B, and PDPN, whereas the most significantly downregulated genes included LINC02283, AC062021.1, AL033519.1, ASIC4-AS1, and SFRP2 (**Figure 5B**).

KEGG enrichment analysis of DEGs revealed distinct functional clustering: upregulated genes in high-risk tumors were primarily enriched in “Phagosome”, “Focal Adhesion”, and “PI3K-Akt Signaling Pathway”, while downregulated genes were associated with “Notch Signaling Pathway” and “TGF-beta Signaling Pathway” (**Figure 5C**). To validate these findings, we performed Gene Set Enrichment Analysis (GSEA) focusing on pathways previously implicated in glioma progression, stratified by risk coefficient. Consistent with GSVA results, high-risk gliomas showed significant activation of “Xenobiotic Metabolism”, “mTORC1 Signaling”, “Epithelial-Mesenchymal Transition”, “DNA Repair”, and “TGF-beta Signaling Pathways” (**Figure 5D**).

Our risk-stratified analysis reveals profound differences in multidimensional signaling pathway activation between glioma risk groups. Notably, high-risk tumors demonstrate coordinated upregulation of oncogenic pathways—including xenobiotic metabolism, mTOR/MAPK signaling, and stress-response cascades—that collectively drive malignant progression.

### 3.6 Genomic aberrations in high- vs low-risk gliomas and validation of core gene expression

#### 3.6.1 Somatic mutation landscapes

**Figure 6:**
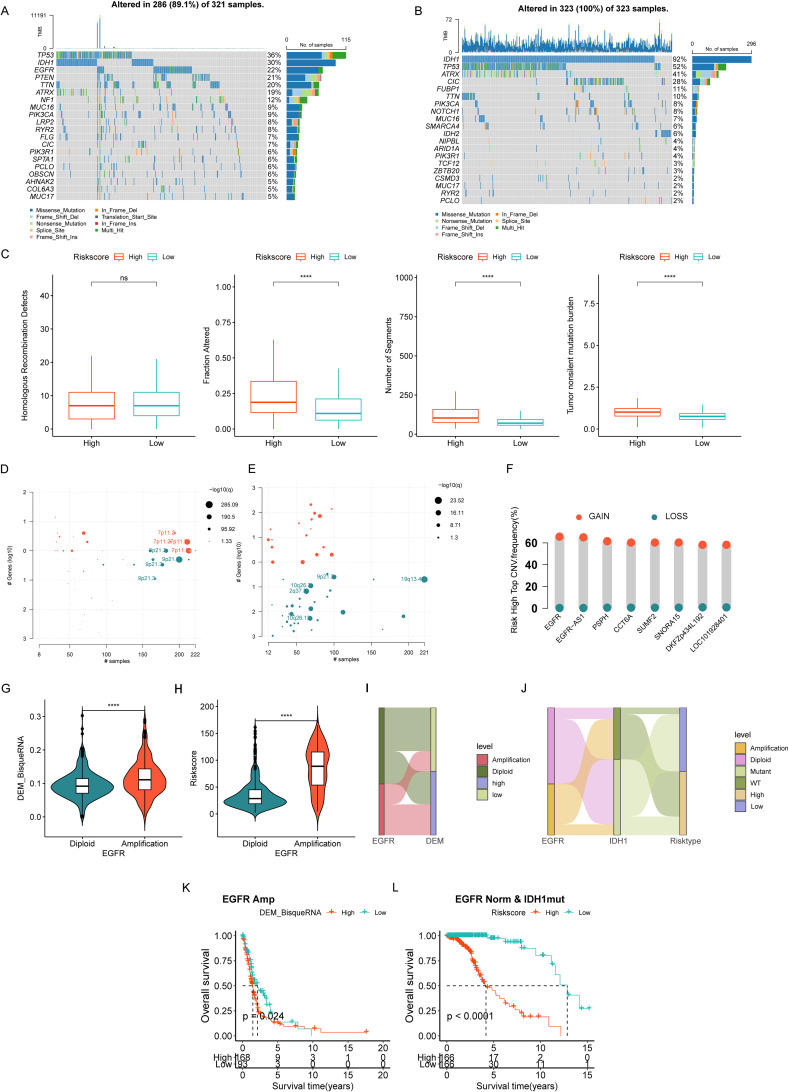
Genetic aberrations between high- and low-risk groups of gliomas. **(A, B)** Mutation waterfall plots depicting mutation profiles of high- **(A)** and low-risk **(B)** glioma subgroups. **(C)** Column graph presenting disparities in homologous recombination defects, fraction alterations, number of segments, and tumor mutation burden between the two subgroups. **(D, E)** Copy number variation landscape plots showing the locus of genomic alteration in the high- **(D)** and low-risk **(E)** subgroups. Red indicates gene amplification; green indicates gene deletion. **(F)** Bar graph presenting the top 8 amplified genes within the 7p11.2 locus in high-risk subgroups. **(G, H)** Comparison of DEM abundances and risk scores between the EGFR amplification group and the diploid group. **(I, J)** Sankey plots visualizing the relationships among EGFR amplification, DEM abundance, IDH1 mutation, and risk score levels. **(K)** Kaplan–Meier curve of overall survival in patients with EGFR amplification according to DEM abundance. **(L)** Kaplan–Meier curve of overall survival in patients with normal EGFR copy number and IDH1 mutation according to high- and low-risk score groups. ****p<0.0001.

Somatic single nucleotide polymorphisms (SNPs) in gliomas are increasingly recognized as key drivers of immunosuppressive tumor microenvironments, primarily through dysregulated immune checkpoint activation and defective antigen presentation (9, 16). To investigate the association between our risk scoring system and somatic mutations, we analyzed mutational profiles of high- and low-risk glioma subgroups using somatic SNP data from the TCGA database (processed via VarScan and the “maftools” package). Genetic alterations were detected in 286/321 (89.1%) high-risk samples and all 323 (100%) low-risk samples. Notably, the top mutated genes in the high-risk group were TP53 (36%), IDH1 (30%), EGFR (22%), PTEN (21%), and TTN (20%), whereas the low-risk group was dominated by mutations in IDH1 (92%), TP53 (52%), ATRX (41%), CIC (28%), and FUBP1 (11%), indicating distinct mutational landscapes between subgroups (**Figures 6A, B**).

Statistical comparisons of mutational characteristics revealed no significant difference in homologous recombination defect (HRD) distribution between risk groups. However, the high-risk cohort exhibited significantly higher values in altered fraction, number of segments, and tumor mutation burden (TMB) compared to the low-risk group (**Figure 6C**).

#### 3.6.2 Copy number variation analysis

CNVs are prevalent in tumors and considered critical drivers of genetic variation (17, 18). Integrated copy number variation (CNV) analysis of TCGA glioma cohorts revealed robust molecular distinctions between risk-stratified groups (**Figures 6D, E**). The high-risk subgroup exhibited recurrent amplification at chromosomal locus 7p11.2 – a genomic region previously associated with enhanced tumor aggressiveness and poor clinical outcomes. Among the top 8 genes within this locus (EGFR, EGFR-AS1, PSPH, CCT6A, SUMF2, SNORA15, DKFZp434L192, and LOC101928401), the proto-oncogene EGFR showed the highest prevalence of copy number gains in high-risk tumors (**Figure 6F**).

Correlation analyses between EGFR copy number status and our risk model revealed that EGFR-amplified samples exhibited significantly higher DEM abundance and risk scores (**Figures 6G, H**). Most EGFR-amplified patients displayed elevated DEM levels (**Figure 6I**), and those with high DEMs had worse overall survival than EGFR-amplified patients with low DEMs (p<0.05) (**Figure 6K**). Consistently, EGFR-amplified patients (regardless of IDH1 mutation status) showed significantly higher risk scores, while most EGFR-nonamplified patients carried IDH1 mutations (**Figure 6J**). Importantly, IDH1-mutant/EGFR-nonamplified patients could be further stratified into low- and high-risk groups using our model, with the high-risk subgroup showing significantly poorer prognosis (p<0.0001) (**Figure 6L**). These findings suggest our model may provide additional risk stratification value for IDH1-mutant gliomas.

#### 3.6.3 Validation of core gene expression in clinical specimens

**Figure 7:**
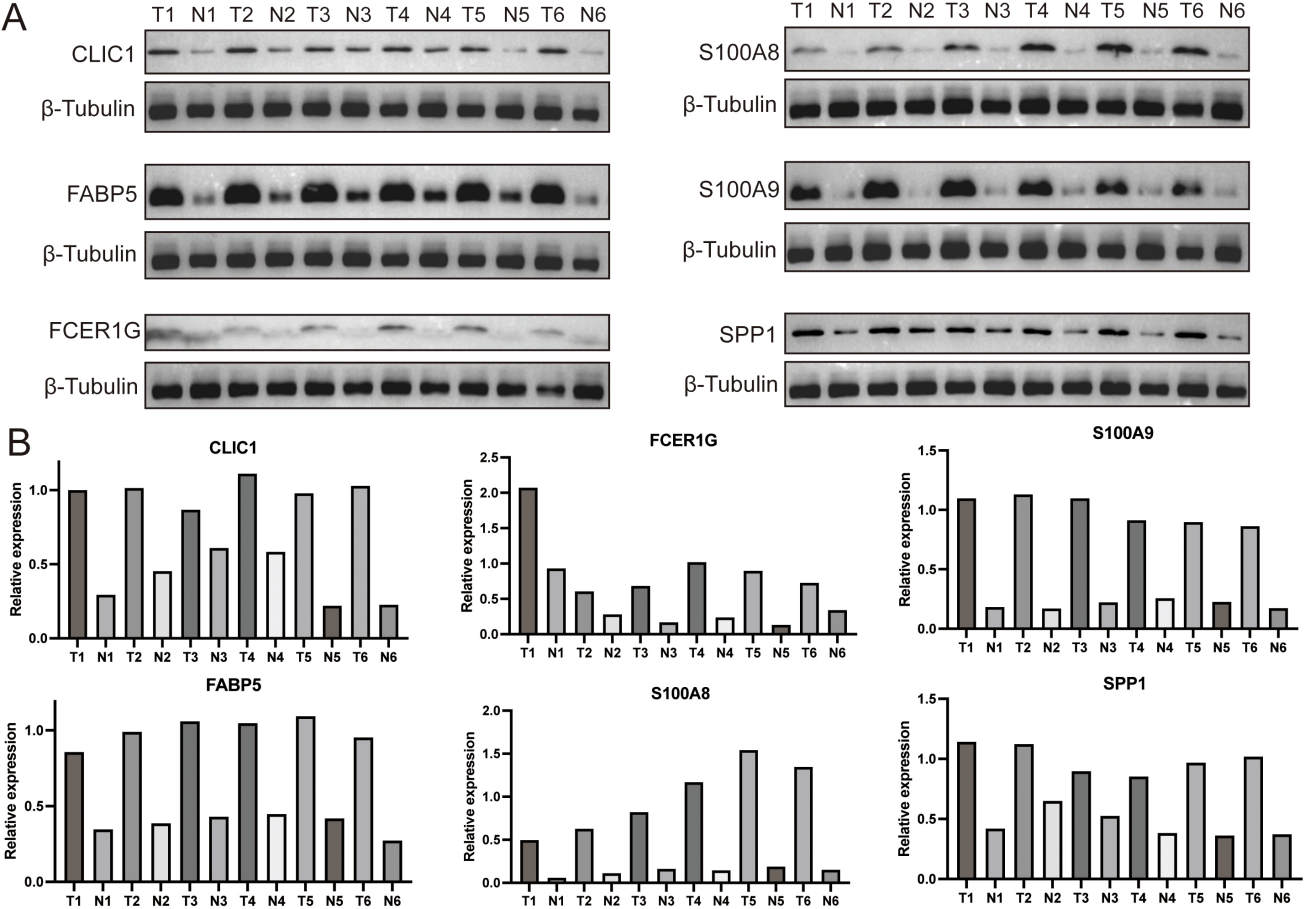
Protein expression validation of core DEM-associated genes in glioblastoma clinical specimens. **(A)** Representative Western blot images showing protein levels of six core DEM-associated genes (CLIC1, FABP5, FCER1G, S100A8, S100A9, SPP1) in glioblastoma tumor tissues (T) versus adjacent normal brain tissues (N). **(B)** Quantitative densitometric analysis of protein expression.

To experimentally validate the biological relevance of our risk model, we collected 6 surgically resected glioblastoma specimens and performed Western blotting (WB) to quantify the expression of 6 core genes (CLIC1, FABP5, FCER1G, S100A8, S100A9, and SPP1) in tumor tissues versus adjacent normal brain tissues. Consistent with our in silico predictions, all 6 proteins were significantly upregulated in glioma tissues compared to normal controls (**Figure 7**), confirming their potential as functional effectors in high-risk glioma progression.

Collectively, our analyses reveal fundamental genomic disparities between glioma risk strata: high-risk tumors exhibit distinct somatic mutation profiles and recurrent 7p11.2 amplification driving EGFR overexpression, while integration of our prognostic model with IDH1 mutation and EGFR amplification status enables synergistic risk stratification—particularly refining prognosis in IDH1-mutant gliomas. Critically, experimental validation confirms significant upregulation of six core effector proteins (CLIC1, FABP5, FCER1G, S100A8, S100A9, and SPP1) in tumor tissues, establishing the model’s biological robustness and nominating actionable targets for molecularly guided therapies.

## 4 Discussion

Traditionally, GAMs are classified into two main phenotypes: classically activated M1 macrophages, which are immunostimulatory and exhibit antitumoral properties, and alternatively activated M2 macrophages, which are immunosuppressive and promote tumor progression (19–21). However, this binary classification oversimplifies the functional heterogeneity and plasticity of GAMs. Recent studies have highlighted the importance of metabolic reprogramming in shaping the functional diversity of macrophages within the tumor microenvironment (22). This study introduces a novel metabolic profiling approach to classify GAMs, providing a more nuanced understanding of their roles in glioma progression.

A key finding of our study is the identification of DEMs from GAMs. Compared with the other three metabolic subtypes, DEMs are enriched in both the metabolism of drugs and xenobiotics and in glycolysis and oxidative phosphorylation (**Figure 1F**), characterized by their high capacity for detoxification and energy-related metabolic processes, which may contribute to the development of drug resistance and the growth or survival of tumor cells (23, 24). Clinically, DEM abundance correlates with advanced tumor grade (**Figure 1G**) and independently predicts poor prognosis, with high-DEM gliomas exhibiting significantly shorter overall survival than low-DEM counterparts (**Figures 2A, B**). Notably, DEMs and GMIMs share functional hallmarks: both exhibit low stemness scores and cluster in late pseudotemporal stages (**Figures 2D, E**). These findings indicate that GAMs may be influenced by the tumor microenvironment after being recruited and develop in the direction of promoting glioma progression. Cytokines such as CC chemokine ligand 2 (CCL2), stromal cell-derived factor 1 (SDF-1), colony stimulating factor 1 (CSF-1), granulocyte–macrophage colony stimulating factor (GM-CSF), tumor necrosis factor (TNF) and glial cell-derived neurotrophic factor (GDNF) promote the immigration of GAMs to the site of the glioma and gradually increase their proportion to 10–50%, eventually resulting in GAMs becoming the primary component of the glioma TME (6, 25). In the tumor microenvironment, factors such as cytokines and hypoxia influence the activation and polarization of TAMs. In turn, activated and polarized TAMs promote tumor invasion, growth, angiogenesis, treatment resistance, and the formation of an immunosuppressive microenvironment (26).

Our integrated multi-omics approach identified six DEM-signature genes (CLIC1, FABP5, FCER1G, S100A8, S100A9, SPP1), validated by Western blotting as upregulated in clinical glioma specimens (**Figure 7**). Among these, S100A8, also known as macrophage migration inhibitor (MIF), was reported to promote TAMs’ evolution from an immune-activated phenotype to an immunosuppressive state through the CD74-MIF axis, which may play an important role in the malignant progression of glioma (27, 28). Additionally, exosome-mediated transfer of MIF enhances TMZ resistance in glioma through downregulating TIMP3 and further activating the PI3K/AKT signaling pathway (29). Secreted phosphoprotein 1 (SPP1) not only serves as an independent prognostic factor for overall survival in glioma patients but also influences macrophage activation, cytokine secretion, and polarization and is strongly associated with various lymphocytes, including T, B and NK cells (30–33). Moreover, SPP1(+) TAMs were validated to be significantly associated with EGFR amplification and impaired T-cell response in grade 2 and 3 gliomas (34). According to the optimal model confirmed by the machine learning integration program based on the DEMs’ characteristic genes, the current study identified differentially expressed genes between the high- and low-risk groups of gliomas, among which CHI3L1, TIMP1 and PDPN were highly expressed in the high-risk group (**Figure 5B**). Chitinase 3-like 1 (CHI3L1) has been reported to facilitate glioma progression and immunosuppression via the NF-kappaB signaling pathway and to modulate the state of glioma stem cells through the CD44-Akt/β-catenin-MAZ loop (35–40). Although the underlying mechanisms have not been thoroughly investigated, TIMP1 and PDPN are also considered to be associated with the malignant progression of gliomas, the formation of an immunosuppressive microenvironment, and even radiotherapy resistance (37, 41–44). In summary, both the characteristic genes of DEMs and the genes highly expressed in the high-risk group determined by the risk prediction model constructed based on these genes play important roles in glioma progression and the formation of the immune microenvironment. These selected genes may underlie the mechanisms by which DEMs are recruited and exert their functions, thus warranting further investigation.

In gliomas, the high immune score, high stromal score, and low tumor purity indicate a high degree of tumor malignancy and poor prognosis (45). According to the prediction model of this study, the risk score was positively correlated with the immune score and stromal score and negatively correlated with tumor purity (**Figure 4B**). M2-type tumor-associated macrophages have been the focus of glioma microenvironment research in recent years and are considered to be closely related to the formation of the immunosuppressive microenvironment and malignant progression of gliomas (32, 46). The results of this study revealed that the infiltration of M2-type macrophages in the high-risk group was significantly greater than that in the low-risk group (**Figure 4A**). Since the M1 and M2 classifications and the metabolic classification are two types of cell classification systems, DEMs are likely to overlap with M2-type macrophages to some extent. A comparative analysis of the high- and low-risk groups revealed that the high-risk group generally presented high expression of immune infiltration and regulatory factors, including CXCR4, CXCL12, CXCL10, CXCL9, and CD40 (**Figure 4C**). Specifically, the CXCR4–CXCL12 axis drives glioblastoma invasion and immunosuppression by recruiting myeloid-derived suppressor cells (MDSCs) and TAMs (47, 48). CXCL9 and CXCL10 exert dual roles in glioblastoma through CXCR3 signaling: while recruiting CD8+ T cells and NK cells to potentiate antitumor immunity, their chronic overexpression paradoxically fosters T-cell exhaustion via sustained IFN-γ signaling and amplifies immunosuppression by facilitating myeloid-derived suppressor cell (MDSC) infiltration (49, 50). This chemokine axis requires precise therapeutic modulation to balance proinflammatory recruitment and exhaustion-resistant T-cell activation. Activation of CD40 enhances antigen-presenting cell (APC) cross-priming and synergizes with PD-1 blockade to overcome T-cell dysfunction. However, its clinical efficacy requires spatiotemporal modulation to avoid protumorigenic NF-κB signaling (51). Among several different algorithms, the number of infiltrating T cells in the high-risk group was inconsistent, whereas the number of infiltrating macrophages consistently increased in the high-risk group (**Figures 4A, D**). Moreover, in the TIP algorithm, the ability of T cells to kill tumor cells in the high-risk group was significantly reduced, which might also be an important reason for the poor prognosis of the high-risk group.

Despite gliomas being characterized by a low tumor mutation burden (TMB) and immunosuppressive features, our study revealed that the high-risk subgroup presented an elevated TMB, an increased fraction of genome alterations (FGAs), and greater numbers of genomic segments than the low-risk subgroup did. Notably, IDH1 mutations were predominantly enriched in the low-risk subgroup (92% vs 30% in high-risk), whereas EGFR mutations ranked as the third most frequent alteration in high-risk gliomas, following TP53 and IDH1 mutations. These findings align with prior evidence demonstrating that IDH-wildtype gliomas harbor greater genomic instability (52). Intriguingly, copy number variation analysis revealed a greater prevalence of EGFR amplification in the high-risk subgroup, with EGFR-amplified tumors exhibiting elevated DEM infiltration and associated risk scores than their EGFR-diploid counterparts. These findings suggest that EGFR amplification may mechanistically drive the recruitment or functional activation of this immunosuppressive macrophage subset, potentially through metabolic reprogramming of the TME. Such EGFR–macrophage crosstalk aligns with prior evidence linking oncogenic EGFR signaling to myeloid cell polarization via STAT3/IDO1 activation (10, 53).

This study has limitations: first, the functional validation of DEMs is constrained by the lack of isolated primary cells from clinical specimens, limiting mechanistic insights into their recruitment and polarization. Second, inconsistencies in immune infiltration estimates across algorithms (**Figures 4A, D**) underscore the need for single-cell resolution studies to dissect DEM-T cell crosstalk. Future work should focus on: (1) isolating DEMs via flow cytometry using our identified surface markers; (2) investigating EGFR-DEM crosstalk in organoid models; and (3) evaluating DEM-targeted therapies (e.g., SPP1/CD44 inhibitors) in preclinical glioma models.

## 5 Conclusions

In conclusion, our findings establish drug metabolism-enriched macrophages (DEMs) as pivotal drivers of immunosuppression and adverse clinical outcomes in gliomas, with machine learning analyses identifying six core DEM-associated genes (CLIC1, FABP5, FCER1G, S100A8, S100A9, SPP1) that form the basis of a robust prognostic model. Mechanistic investigations revealed high-risk gliomas exhibit paradoxical immune infiltration characterized by impaired T-cell cytotoxicity, concurrent with hyperactivated oncogenic signaling and EGFR amplification, while integration of the DEM-derived risk signature with EGFR/IDH status yields a clinically actionable framework for prognostication. Notably, Western blot validation confirmed protein-level upregulation of all six core genes in glioblastoma tumor tissues relative to adjacent normal brain tissues, reinforcing their functional relevance and potential as therapeutic targets. Collectively, these results uncover a novel metabolic-immune crosstalk mediated by DEMs, providing critical insights for developing precision immunotherapies targeting glioma-associated macrophage plasticity.

## Data Availability

The datasets presented in this study can be found in online repositories. The names of the repository/repositories and accession number(s) can be found in the article/**Supplementary Material**.
