# Supplementary material for: Metabolic reprogramming of glioma-associated macrophages identifies detoxification and energetic macrophages as drivers of immunosuppression and therapeutic vulnerability
Source: Front Immunol. 2026 Feb 11;17:1752553. doi: 10.3389/fimmu.2026.1752553 (PMC12932590; doi:10.3389/fimmu.2026.1752553)
Supplement: Supplementary file 1 [file DataSheet1.docx]

Supplementary Material

## Supplementary Figures


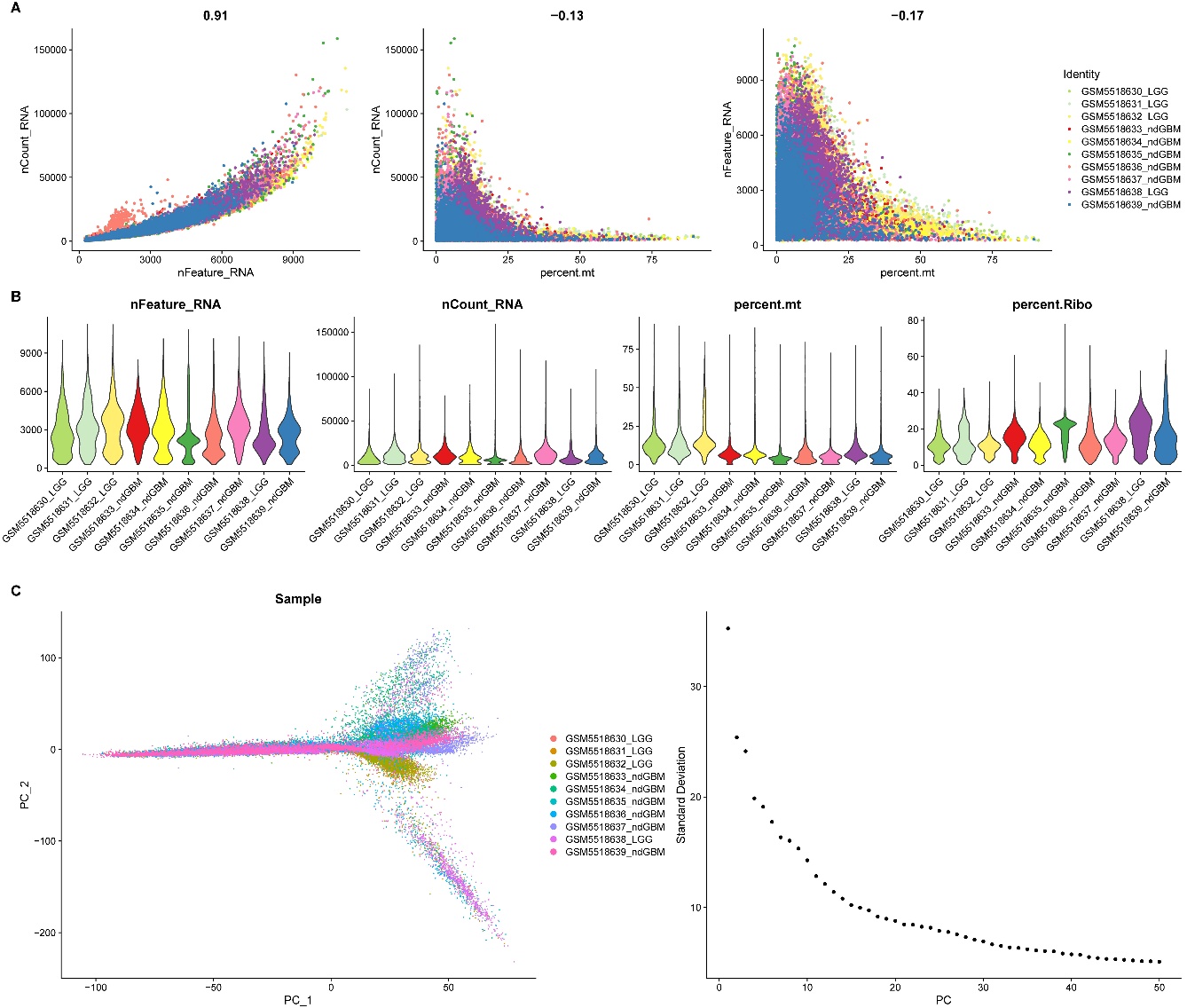


**Supplementary Figure 1.** Cell quality control, normalization, and PCA reduction of the single-cell dataset. (A) Scatter plots illustrating the correlations between nCount_RNA and nFeature_RNA (correlation coefficient = 0.91), between nCount_RNA and percent.mt (correlation coefficient = -0.13), and between nCount_RNA and percent. Ribo (correlation coefficient = -0.17). (B) Violin plots depicting the distributions of nFeature_RNA, nCount_RNA, [percent.mt](http://percent.mt), and percent. Ribo across different samples. (C) PCA plots showing the distribution of samples after quality control in the principal component space. Different colors represent different samples, and the standard deviation curve indicates the degree to which each principal component explains the overall variance.


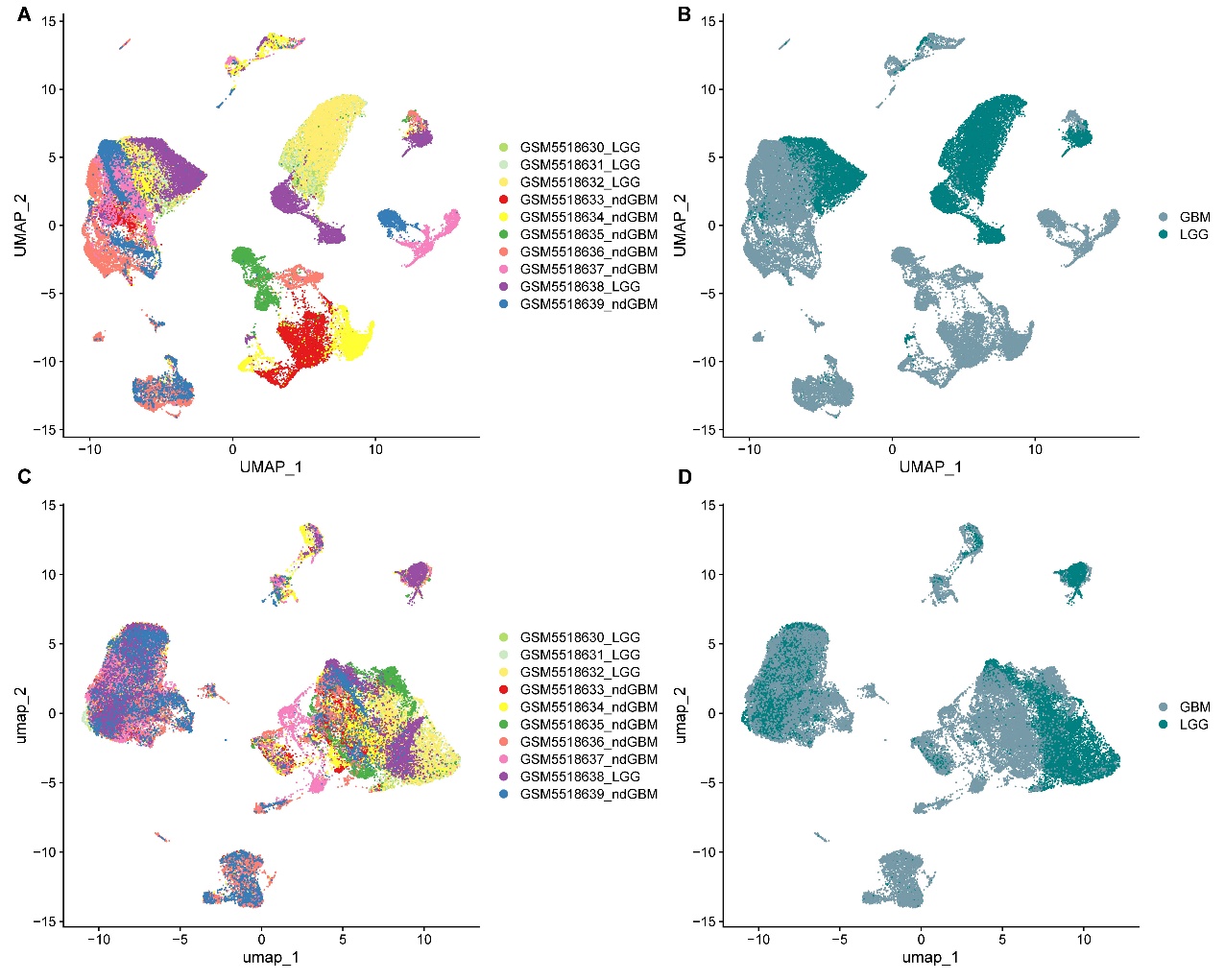


**Supplementary Figure 2.** UMAP visualization of samples before and after batch removal. (A, B) UMAP visualization of the raw data. (C, D) UMAP visualization after batch effect removal.


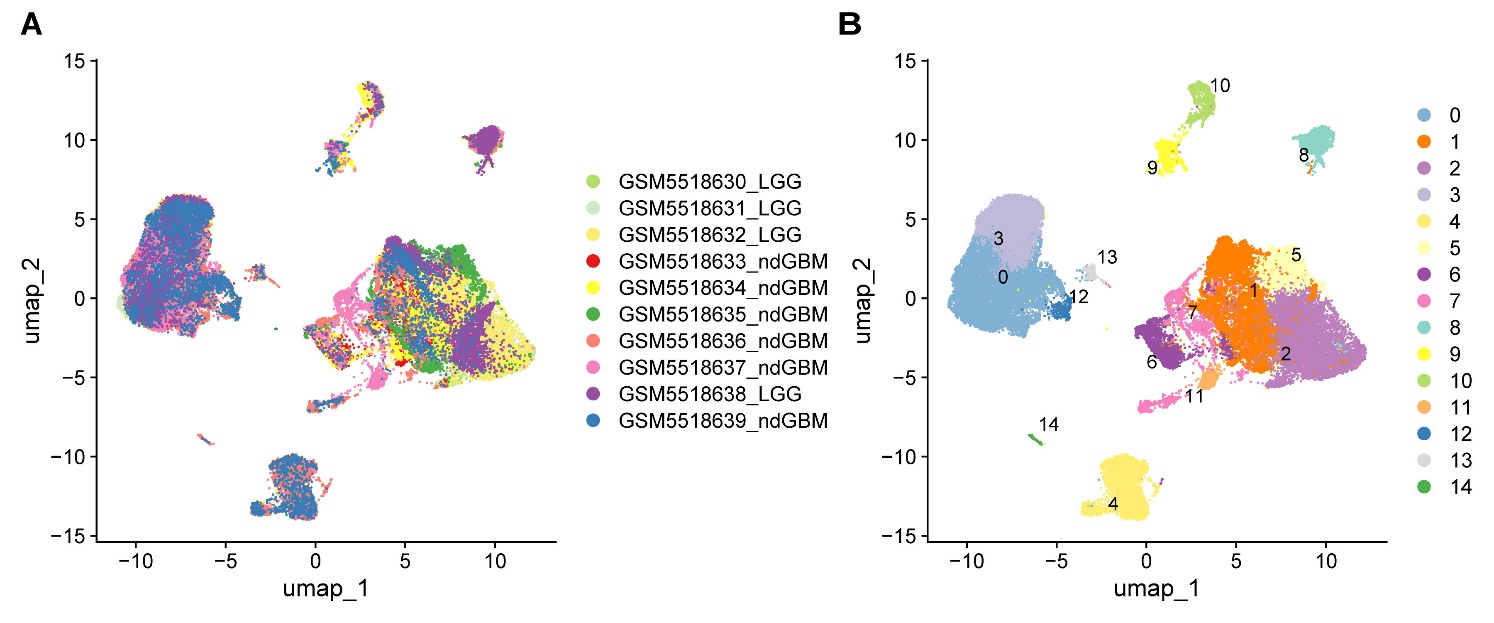


**Supplementary Figure 3.** Visualization of UMAP-based cell clustering and subpopulation distribution. Each point represents a cell, with different colors for different samples (A) or different subpopulations (B).


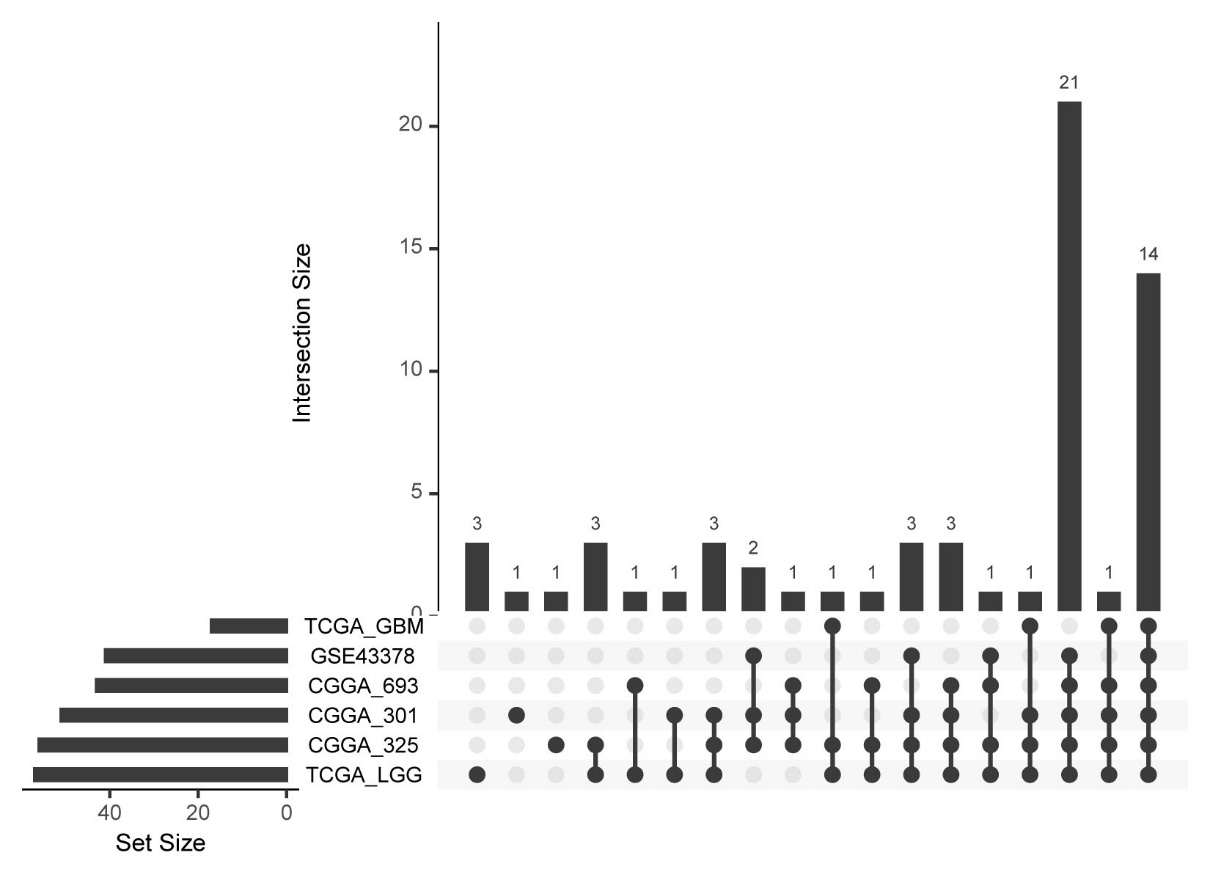


**Supplementary Figure 4.** Gene screening for the glioma prognostic model via univariate Cox analysis in multiple datasets. The scatter plot represents the number of unique genes in each dataset. The gray dots indicate genes whose expression did not reach the level of significance, whereas the black dots represent genes whose expression was significantly different.
